# The Role of Genetic Sex in Affect Regulation and Expression of GABA-Related Genes Across Species

**DOI:** 10.3389/fpsyt.2013.00104

**Published:** 2013-09-17

**Authors:** Marianne L. Seney, Lun-Ching Chang, Hyunjung Oh, Xingbin Wang, George C. Tseng, David A. Lewis, Etienne Sibille

**Affiliations:** ^1^Department of Psychiatry, University of Pittsburgh, Pittsburgh, PA, USA; ^2^Translational Neuroscience Program, University of Pittsburgh, Pittsburgh, PA, USA; ^3^Department of Biostatistics, University of Pittsburgh, Pittsburgh, PA, USA; ^4^Center for Neuroscience, University of Pittsburgh, Pittsburgh, PA, USA; ^5^Department of Human Genetics, University of Pittsburgh, Pittsburgh, PA, USA; ^6^Department of Computational and Systems Biology, University of Pittsburgh, Pittsburgh, PA, USA

**Keywords:** GABA, genetic sex, mood, somatostatin, anxiety, depression

## Abstract

Although circulating hormones and inhibitory gamma-aminobutyric acid (GABA)-related factors are known to affect mood, considerable knowledge gaps persist for biological mechanisms underlying the female bias in mood disorders. Here, we combine human and mouse studies to investigate sexual dimorphism in the GABA system in the context of major depressive disorder (MDD) and then use a genetic model to dissect the role of sex-related factors in GABA-related gene expression and anxiety-/depressive-like behaviors in mice. First, using meta-analysis of gene array data in human postmortem brain (*N* = 51 MDD subjects, 50 controls), we show that the previously reported down-regulation in MDD of somatostatin (*SST*), a marker of a GABA neuron subtype, is significantly greater in women with MDD. Second, using gene co-expression network analysis in control human subjects (*N* = 214; two frontal cortex regions) and expression quantitative trait loci mapping (*N* = 170 subjects), we show that expression of *SST* and the GABA-synthesizing enzymes glutamate decarboxylase 67 (*GAD67*) and *GAD65* are tightly co-regulated and influenced by X-chromosome genetic polymorphisms. Third, using a rodent genetic model [Four Core Genotypes (FCG) mice], in which genetic and gonadal sex are artificially dissociated (*N* ≥ 12/group), we show that genetic sex (i.e., X/Y-chromosome) influences both gene expression (lower *Sst*, *Gad67*, *Gad65* in XY mice) and anxiety-like behaviors (higher in XY mice). This suggests that in an intact male animal, the observed behavior represents the outcomes of male genetic sex increasing and male-like testosterone decreasing anxiety-like behaviors. Gonadal sex was the only factor influencing depressive-like behavior (gonadal males < gonadal females). Collectively, these combined human and mouse studies provide mechanistic insight into sexual dimorphism in mood disorders, and specifically demonstrate an unexpected role of male-like factors (XY genetic sex) on GABA-related genes and anxiety-like behaviors.

## Introduction

Major depressive disorder (MDD) and anxiety disorders are devastating, often chronic illnesses of altered mood regulation. These disorders affect a significant portion of the population, with a projected lifetime risk of ∼23% for MDD and ∼32% for an anxiety disorder (1). Although MDD and anxiety disorders are different diagnoses, they are included in the broader definition of mood disorders and findings from large-scale epidemiological studies across cultures show high comorbidity and co-segregation within the symptom dimension of high negative affect (i.e., internalization) (2–4). MDD and anxiety disorders also share common genetic risk (5), and symptom remission is observed in both conditions after chronic treatment with selective serotonin reuptake inhibitors, together suggesting the presence of common contributing biological factors. Women are twice as likely to be diagnosed with MDD or an anxiety disorder, and anxiety symptoms are almost always co-morbid with MDD in women, making the two difficult to separate.

Adult hormone levels are linked to mood states, particularly in relation to premenstrual dysphoric disorder and postpartum depression (6). Rodent studies report links between circulating hormone levels (“activational” hormone effects) and anxiety-/depressive-like behaviors (7), although systematic reviews of human studies do not report consistent associations (8). MDD prevalence is higher in women across life stages and hormonal states, indicating that structural or biological differences other than adult circulating hormones place women at higher risk. Indeed, permanent sexual dimorphism of the brain is established by gonadal hormone exposure during critical developmental periods (“organizational” effects) and sex chromosome effects (“genetic” sex). Thus, sexual dimorphism in mood disorders could be due to (i) activational effects of hormones in adulthood, (ii) organizational effects of hormones during development (due to different gonadal sex), (iii) differences in genetic sex, and/or (iv) a combination of the above factors. We recently reported a partial contribution of developmental hormone exposure to sexual dimorphism in adult anxiety-/depressive-like behavior (referred to as behavioral emotionality) in mice (9), suggesting that an alternative sex-related mechanism, potentially genetic sex, also influences emotionality. Even though genetic sex has a predominant role during development in determining gonadal sex, contributions of X/Y-linked genes are ongoing throughout life.

Evidence from neuroimaging and postmortem neuroanatomical and molecular studies suggest dysfunction in corticolimbic emotion regulation centers of the brain in mood disorders (10). This corticolimbic network includes the dorsolateral prefrontal cortex (DLPFC), anterior cingulate cortex (ACC), hippocampus, ventral striatum, anterior thalamic nuclei, and amygdala (10, 11). Magnetic resonance spectroscopy studies reported decreased cortical gamma-aminobutyric acid (GABA) content in MDD (12) and correlation with emotional processing (13), while molecular studies further suggest reduced GABA-mediated inhibition (14–16). Additionally, drugs that potentiate GABA function (e.g., benzodiazepines) are anxiolytic (17). Together these studies support the hypothesis of a deficit in inhibitory neurotransmission in MDD, potentially related to low mood. These human findings are supported by causal studies in mice, where mild reduction in GABA-signaling is sufficient to induce anxiety-/depressive-like behaviors (18). Interestingly, many studies have suggested that hormones modulate mood by altering GABA transmission (19), suggesting a potential link between sexual dimorphism in GABA function and in risk for mood disorders. In human MDD subjects, the amygdala (16), ACC (20, 21), and DLPFC (22) exhibit lower markers of GABA interneurons, especially somatostatin (*SST*), a marker for the subtype of GABA neurons that target the dendritic compartment of pyramidal cells. Notably, reports of low *SST* in MDD are often more robust in females (16, 21). In concert, these findings suggest a GABA/*SST*-related cellular phenotype of reduced dendritic inhibition in depression.

We tested here the hypothesis that genetic sex influences sexual dimorphism in emotionality and associated gene expression. Specifically, using both human postmortem brains and a mouse model which can dissociate the relative contributions of gonadal and genetic sex, we examined the possibility that genetic sex controls expression of GABA-related genes and associated anxiety- and depressive-like behaviors.

## Materials and Methods

### Human subjects

Brain samples were obtained after consent from next-of-kin during autopsies conducted at the Allegheny County Medical Examiner’s Office (Pittsburgh, USA) using procedures approved by University of Pittsburgh’s Institutional Review Board and Committee for Oversight of Research Involving the Dead. Consensus DSM-IV diagnoses were made by an independent committee of experienced clinical research scientists using information from clinical records, toxicology results, and a standardized psychological autopsy (23).

### Meta-analysis of *SST* gene expression in MDD datasets

#### Subjects

A total of 51 subjects with MDD and 50 control subjects were included in the eight studies. DLPFC, subgenual ACC (sgACC), or rostral amygdala samples enriched in lateral, basolateral, and basomedian nuclei had been previously collected. Two studies were performed in DLPFC, four studies in sgACC, and two studies in amygdala. Half of the studies had been performed in female subjects in each brain region. Details on all subjects, areas investigated, and subject parameters are available in Table 1. Group means for age, postmortem interval (PMI), RNA integrity number (RIN; measured by Agilent Bioanalyzer; Santa Clara, CA, USA), and brain pH were nearly identical and not statistically different for all studies. Details on each cohort were previously reported [DLPFC (22), sgACC (21), amygdala (16)].

**Table 1:** **Data description of eight MDD microarray studies used for meta-analysis of *SST* results**.

Study name	Sex	Brain region	Sample size	Array platform	Covariates for *SST*
MD2_DLPFC_M	Male	DLPFC	32 (16 pairs)	Affy. HG-U133 Plus 2	Age, PMI
MD1_ACC_M	Male	ACC	30 (15 pairs)	Affy. HG-U133 Plus 2	Age
MD2_ACC_M	Male	ACC	18 (9 pairs)	Affy. HG-U133 Plus 2	None
MD1_AMY_M	Male	AMY	28 (14 pairs)	Affy. HG-U133 Plus 2	Age
MD2_DLPFC_F	Female	DLPFC	28 (14 pairs)	Affy. HG-U133 Plus 2	Age, PMI
MD3_ACC_F	Female	ACC	28 (14 pairs)	Illumina HumanHT-12	Age, RIN
MD2_ACC_F	Female	ACC	22 (11 pairs)	Illumina HumanHT-12	Alcohol, suicide
MD3_AMY_F	Female	AMY	42 (21 pairs)	Illumina HumanHT-12	Age, suicide

ACC, anterior cingulate cortex; AMY, amygdala; DLPFC, dorsolateral prefrontal cortex; PMI, postmortem interval; RIN, RNA integrity number. Note that some subjects are included in multiple studies (i.e., more than one brain region investigated for some subjects). We accounted for this putative dependence structure by keeping the same permutation order for each pair of studies of the same cohort in the permutation analysis of individual studies.

#### Quantitative real-time PCR validation of microarray meta-analysis

Quantitative real-time PCR (qPCR) results for *SST* from individual published studies on the DLPFC (22), sgACC (20, 21), amygdala (16) were combined by Stouffer’s Z-trend meta-analysis. Briefly, mean *SST* expression in control subjects was set at 100% expression, and mean *SST* expression in MDD subjects was expressed as a percentage of control expression. To calculate the mean and standard error for *SST* across areas, the weighted averages (based on *N* in each study) for each study were combined. Meta P software (24) was then used to combine *p*-values for each individual study. Stouffer’s Z-trend *p*-values are reported, which take into account *p*-values, the sample size of each individual study and the direction of effect.

### Gene networks and expression quantitative trait loci

#### Subjects

A total of 214 control individuals (no DSM-IV diagnosis) were used in this study (see Table 2 for technical variables of cohort). Upon brain collection, ∼2 cm coronal blocks from the right hemisphere were cut through the rostro-caudal extent of the brain and stored at −80°C. The RIN of each brain was assessed by chromatography (Agilent Bioanalyzer; Santa Clara, CA, USA). Samples were obtained from two prefrontal cortex regions: Brodmann areas (BA) 11 and 47. Detailed information on gene arrays, expression measures, and genotyping is included in Appendix.

**Table 2:** **Technical variables for human postmortem brains used in QTL and gene network analyses**.

Variable	*N* (%) or mean (SD)
Age, years	50.8 (14.9)
Range	16–96
Sex
Male	167 (79)
Female	44 (21)
Race
Caucasian	180 (85)
African-American	31 (15)
PMI	17.2 (5.9)
Range	4.8–37.5
pH	6.7 (0.3)
Range	5.8–7.6
RIN	8.0 (0.73)
Range	5.9–9.6

PMI, postmortem interval; RIN, RNA integrity number.

#### Functional and network analysis using Ingenuity Pathway Analysis

For BA11 and BA47 separately, Pearson correlation values were calculated between *SST* and each transcript examined by microarray. These correlation with *SST* values were highly similar across regions (*R* > 0.95), thus the brain region-specific correlation values were averaged to get correlation with *SST* across brain regions. Analysis was performed for the top 200 genes positively correlated with *SST* (*R* > 0.725) in the gene expression dataset from 214 control subjects (no DSM-IV diagnosis; BA11, BA47), to investigate biological pathways potentially affected by *SST*. Genes selected were overlaid onto the global molecular network of ingenuity pathway analysis (IPA) (Ingenuity^®^ Systems, www.ingenuity.com), a database of over 3.5 million literature-based links between genes and other bioactive molecules. IPA^©^ creates networks based on literature-based evidence for functional links between genes.

#### GABA-signaling-related gene network

Genes related to GABA-signaling (e.g., GABA receptor subunits, markers of GABA neurons) were selected to build a weighted gene co-expression network. Two genes or transcripts are considered co-expressed if their patterns of expression are correlated across samples; this link has been shown to reflect shared function, through multiple potential biological pathways, including common regulatory pathways (e.g., hormone signaling, transcription factors) (25). Here, we only considered expression data from BA11. Pearson correlation values were calculated between individual GABA-signaling-related gene and every other GABA-signaling-related gene to create a co-expression table. Pearson correlation values greater than 0.5 were then used to create a weighted gene co-expression network that was visualized in Cytoscape (2.8.3).

#### Trans-eQTL

We focused on genetic variation on the X-chromosome to identify single nucleotide polymorphisms (SNPs) associated with expression of *SST* and GABA-synthesizing enzymes glutamate decarboxylase 67 (*GAD67*) and *GAD65*; since these genes are not on the X-chromosome, this is considered *trans*-expression quantitative trait loci mapping. We used samples from white subjects here (*N* = 170; 136 males, 34 females; males and females analyzed separately). Detailed methods for trans-eQTL mapping are included in Appendix.

#### *Cis*-eQTL (association of X-chromosome SNPs with nearby genes)

We then performed cis-eQTL analyses to identify SNPs that are associated with expression of nearby genes (within 50 kb of genes). The same samples were used here as for the trans-eQTL studies (*N* = 170 white subjects; 136 males and 34 females). SNPs were selected for this analysis only if they were previously identified as having significant association with *SST*, *GAD67*, and/or *GAD65*. In the ANCOVA model, we adjusted for age, brain pH, and RNA integrity (RIN values), as these three covariates significantly influenced gene expression in both BA11 and BA47, and detected whether the identified SNPs altered expression of any nearby transcripts (performed in males and females separately). We did not control for multiple testing, as we had *a priori* hypotheses that these SNPs would alter gene expression. Statistical significance was set at *p* < 0.05.

### Mice

Four Core Genotypes (FCG; Jackson Labs, Bar Harbor, ME, USA; B6.Cg-Tg(Sry)2Ei *Sry^dl1Rlb^*/ArnoJ) mice were used (26). By crossing a C57BL/6J female with an XY^−^*Sry* male (Y^−^ denotes absence of the testis-determining gene, *Sry*, on the Y-chromosome; *Sry* denotes presence of the autosomal testis-determining *Sry* transgene), four groups of mice are generated: XX gonadal males (XX*Sry*), XX gonadal females (XX), XY^−^ gonadal males (XY^−^*Sry*), and XY^−^ gonadal females (XY^−^). Each breeding pair can generate all four genotypes, eliminating any possible litter effects. Due to the genetic manipulation, cages of gonadal females contained both XX and XY^−^ mice, and cages of gonadal males contained both XX*Sry* and XY^−^*Sry* mice. All mice were maintained in group housing (separated by gonadal sex) under standard conditions (12/12 h light/dark cycle, 22 ± 1°C, food and water *ad libitum*; same room) in a pathogen free animal facility, in accordance with the University of Pittsburgh Institutional Animal Care and Use Committee. Note that cages contained standard bedding material, with no additional enrichment. The University of Pittsburgh is fully accredited by the American Association for Accreditation of Laboratory Animal Care (AAALAC).

### Experimental design for mouse studies

**Figure 1:**
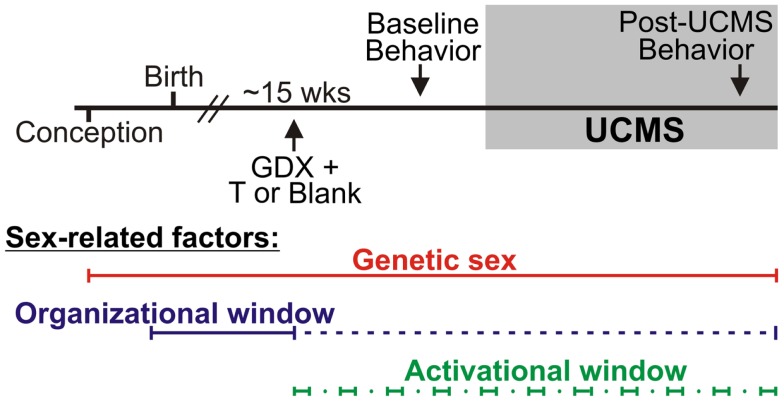
**Experimental design and sex-related factors**. FCG mice were gonadectomized (GDX) at approximately 15 weeks old and implanted with either testosterone (T)-filled or blank capsules. After allowing 3 weeks for animals to recover from surgery, all mice were exposed to behavioral tests to assess baseline (“non-stress”) anxiety-like behavior and activity. Mice were then exposed to 7 weeks of unpredictable chronic mild stress (UCMS). During weeks 6 and 7 of UCMS, mice were exposed to behavioral tests to assess chronic stress-induced anxiety-like and anhedonia-/depressive-like behavior and activity. Sex-related factors are shown at the bottom to indicate windows of time in which genetic sex, gonadal sex, and/or circulating hormones can act.

Refer to Figure 1. Numbers of mice per group are in Table 3 (*N* = 50–60 mice/main factor). FCG mice were un-manipulated until gonadectomized (GDX) at 15 weeks. Half the mice from each genotype were implanted with testosterone-filled capsules and half with blank capsules (to investigate activational effects of circulating testosterone); cages of group-housed mice were randomly assigned to receive either blank or testosterone-filled capsules so as to create similar numbers of blank and testosterone-treated mice for each genotype. After allowing 3 weeks for hormone levels to equilibrate, mice were tested in the elevated plus maze (EPM) and open field (OF) to investigate baseline anxiety-like behaviors. All mice were then exposed to 7 weeks of unpredictable chronic mild stress (UCMS), a paradigm that robustly increases anxious/depressive-like behaviors, thus presenting homologous features to human depressive-like symptoms (27). During UCMS weeks 5–7, animals were exposed to the EPM and OF to investigate stress-induced anxiety-like behaviors, and sucrose preference for anhedonia-/depressive-like behavior. During all behavior testing, experimenters were blind to genotype and hormone-treatment groups. A longitudinal study (i.e., baseline behavior followed by UCMS exposure followed by post-UCMS behavior) was employed due to experimental constraints on the number of animals that can be used in a UCMS/behavior study, since the focus of the experimental contrasts were on group differences under baseline and high emotionality states. The EPM, OF, and sucrose preference tests were selected to examine anxiety- and depressive-like behavior in mice. The pharmacological validation of these tests using known antidepressant and anxiolytic compounds has been extensively characterized [reviewed in (28–30)]. Mice were sacrificed and brains used for real-time quantitative PCR analysis of *Sst*, *Gad67*, and *Gad65* gene expression.

**Table 3:** **Treatment and size of experimental groups**.

Genotype	Geneticsex	Gonadalsex	Activationalgroup	Baseline*N*	Post-UCMS*N*
XX	Female	Female	GDX + blank	14	13
			GDX + T	20	20
XX*Sry*	Female	Male	GDX + blank	17	17
			GDX + T	15	14
XY^−^	Male	Female	GDX + blank	13	13
			GDX + T	13	13
XY^−^*Sry*	Male	Male	GDX + blank	12	12
			GDX + T	15	14

GDX, gonadectomy; T, testosterone.

### Gonadectomy and hormone-treatment

Under isoflurane anesthesia, 15-week-old mice were bilaterally GDX to remove endogenous, gonadal sources of hormones. GDX animals received a subcutaneous SILASTIC (Dow Corning Corp., Midland, MI, USA) capsule containing 5 mm crystalline testosterone (1.57 mm ID × 2.41 mm OD), while the other half received blank capsules (*N* = 12–20 mice/group; *N* = 50–63/main effect). This size testosterone capsule yields circulating testosterone levels at or slightly above normal male levels. At the time of sacrifice, we collected trunk blood for testosterone assay to confirm the efficacy of our adult hormone manipulation. Serum samples were sent to the University of Virginia Center for Research in Reproduction Ligand Assay and Analysis Core (supported by the Eunice Kennedy Shriver NICHD/HIH (SCCPIR) Grant U54-HD28934) and RIA was used to determine testosterone concentration. Groups treated with testosterone capsules did not differ in serum testosterone levels (*p* > 0.4).

### Unpredictable chronic mild stress

UCMS replicates the role of stress in eliciting MDD, models several MDD symptom dimensions, and respects the timeframe of onset and efficacy of antidepressant treatment (29). Briefly, the UCMS protocol consisted of a 7-week period during which group-housed mice were exposed to a randomized schedule of environmental disturbances approximately 1–2 times per day, 7 days a week, as applied in our lab (29, 31, 32). Disturbances included forced bath (∼2 cm of water for 15 min), aversive smell (1 h exposure to bobcat urine), light cycle reversal or disruption, social stress (rotate mice into previously occupied cages), tilted cage (45°tilt), mild restraint (50 ml conical tube with air hole for 15 min), bedding change (replace soiled bedding with clean bedding), wet bedding, and no bedding. Weekly assessment of weight and fur were performed to track progression of the UCMS syndrome [as in (29)].

### Behavioral testing

#### Elevated plus maze

Behavior in the EPM was measured during the light phase as previously described (33) using a cross maze with 2 open and 2 closed 30 cm × 5 cm arms. Time spent and percent entries (entries into open arms divided by entries into open or closed arm × 100) in the open arms were recorded for 10 min to measure anxiety-like behavior. The total number of entries into any arm was used as an index of locomotor behavior.

#### Open field

The OF test was performed during the light phase in a 50.8 cm × 50.8 cm arena, and the center of the OF was defined as the centermost 25.4 cm × 25.4 cm of the arena. ANY-Maze software (Stoelting; Wood Dale, IL, USA) was used to track behavior that was recorded using a ceiling-mounted video camera. The time spent and percent distance (distance in center divided by total distance × 100) in the center of the arena were recorded for 10 min as measures of anxiety-like behavior. The total distance traveled was recorded as an index of locomotor activity.

#### Sucrose preference

During training for sucrose preference, group-housed mice have free access to both water and a 2% sucrose solution for 48 h to reduce neophobia. After the initial 24 h of training, the orientation of the sucrose and water bottles is switched so that the mice do not learn to associate the sucrose with a particular location relative to the water filled bottle. Following training, mice were single-housed in a new cage for 16 h (during the dark phase) and given free access to both water and 2% sucrose; amount water and amount sucrose were recorded. Sucrose intake as a percentage of total volume of fluid consumed denotes sucrose preference. Due to experimental constraints, only half the mice from each group (*n* = 60 total) were exposed to the sucrose preference test.

### Frontal cortex dissection gene expression analysis

Brains from the mice used in behavioral analysis were flash frozen on dry ice at the time of sacrifice (after 7 weeks UCMS and while still being exposed to stressors) and stored at −80°C. One-hundred and sixty micrometer thick rostro-caudal sections were obtained using a cryostat and a 1 mm bore tissue punch was used to isolate the frontal cortex [cingulate cortex and prelimbic cortex; between Bregma +2.34 and +0.50 mm; (34)]. cDNAs were generated from RNA extracted from frontal cortex tissue punches. Using qPCR we examined the expression of three genes associated with cortical GABA microcircuitry: two genes coding for GABA-synthesizing enzymes (*Gad67*, *Gad65*), and *Sst*, a marker of dendritic-targeting GABA neurons. Detailed information on qPCR methods is included in Appendix.

### Statistical analysis

As previously described for FCG mice (35), we used three-way ANOVA (gonadal sex-by-adult testosterone-treatment-by-genetic sex) to compare groups for each dependent measure (behavioral, gene expression). The genetic sex effects were examined by comparing XX to XY^−^ mice. Gonadal sex effects were analyzed by comparing mice with ovaries (XX, XY^−^) to those with testes (XX*Sry*, XY^−^*Sry*); since all mice were GDX several weeks prior to behavioral testing, any differences observed due to gonads are considered to be organizational effects (permanent changes due to hormone exposure during a critical developmental period). To examine effects of adult testosterone exposure, we compared mice GDX in adulthood and implanted with testosterone-filled capsules to those GDX and implanted with blank capsules; these are considered to be activational effects of testosterone. If the three-way ANOVA was significant for any main effect or interaction, we performed planned contrasts using Tukey’s *post hoc* test. Data are expressed as mean ± SEM and statistical significance was set at *p* < 0.05, and trend-level at *p* < 0.1.

## Results

### Sex-specific reduction of *SST* in major depression

**Figure 2:**
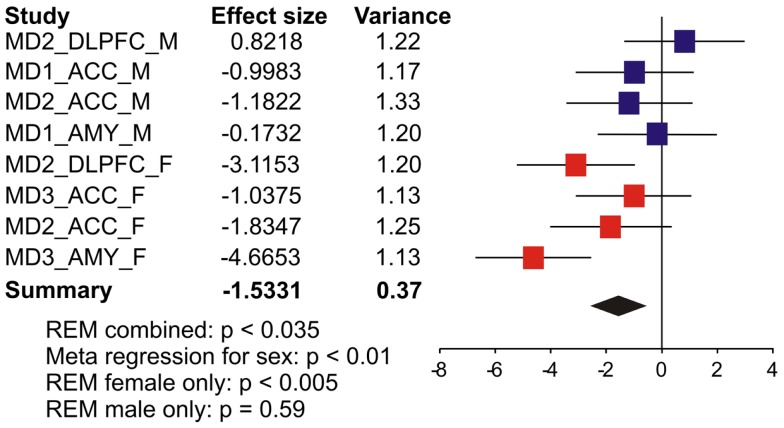
**Somatostatin (SST) expression is more robustly down-regulated in women with MDD**. Results from eight microarray studies [MDD subjects (*N* = 52) versus controls (*N* = 51)] were combined by meta-analysis. Two studies were performed in dorsolateral prefrontal cortex (DLPFC), four in anterior cingulate cortex (ACC), and two in amygdala (AMY). Half of the studies were performed in female subjects in each region. *X*-axis indicates effect of MDD. The left-hand columns list the names of the studies and the effect size values. The summary effect of each study is depicted as a point estimate (mean effect size) bounded by its 95% confidence interval (±1.96 standard deviation), represented by horizontal lines. The summarized effect is plotted as a diamond, which is shown on the bottom line with the associated REM *p*-value. The lack of overlap with the vertical no-effect line (effect size = 0) for the summarized diamond indicates statistical significance of the meta-analysis.

**Figure 3:**
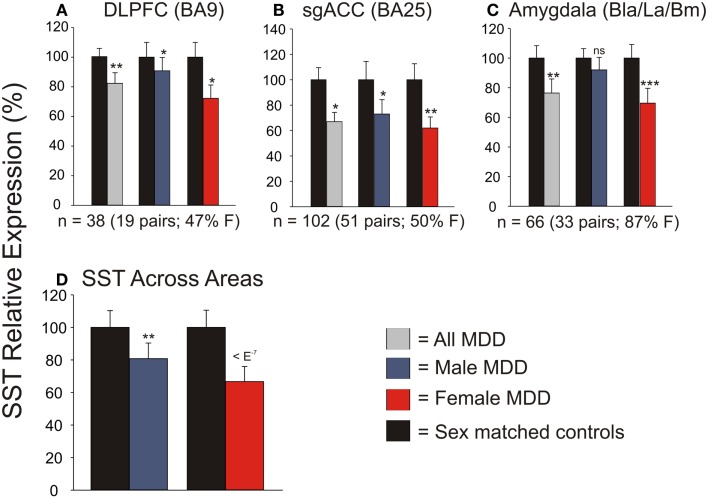
**qPCR validation of microarray meta-analysis**. Individual qPCR results in the dorsolateral prefrontal cortex (DLPFC) **(A)**, subgenual anterior cingulate cortex **(B)**, and amygdala **(C)**. **(D)** When individual qPCR results are combined by meta-analysis, females have a much more robust down-regulation of SST expression than males. *p < 0.05; **p < 0.01; ***p < 0.001; ns, not significant.

We first systematically investigated prior reports *suggesting* a greater down-regulation of *SST* in female MDD (21). Using meta-analysis of eight human postmortem microarray studies across corticolimbic brain regions (DLPFC, ACC, AMY; Table 1) [see (36, 37)], we *confirmed* that *SST* was decreased in subjects with MDD compared to controls; however when males and females were considered separately, the *SST* reduction was significant in females, but not males. Using meta-regression, we *confirmed* that the low *SST* findings in female MDD was significantly different from results in male MDD (Figure 2), together demonstrating a sexual dimorphism in reduced *SST* in MDD. We confirmed these results with meta-analysis of *SST* qPCR results from multiple studies, across multiple brain regions. In the DLPFC (Figure 3A), *SST* is significantly down-regulated in males and females separately, as well as when males and females are combined. In the sgACC (Figure 3B) *SST* is significantly down-regulated in males and females separately, as well as when males and females are combined. In the amygdala (Figure 3C), *SST* is significantly down-regulated in females, but not males. When qPCR results are combined by meta-analysis across areas (Figure 3D), the *SST* down-regulation is more robust in females (−33%) than in males (−19%).

### *SST*-based gene network analysis in human frontal cortex

**Figure 4:**
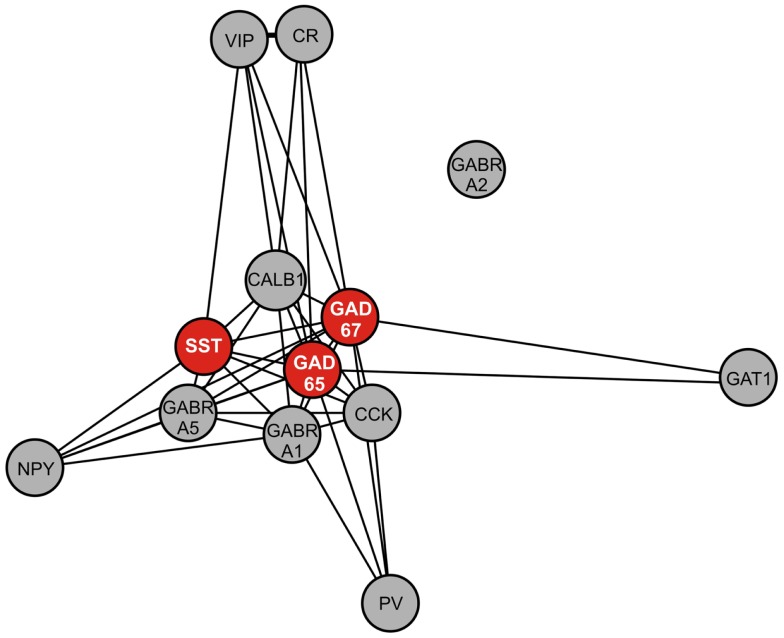
**Weighted gene co-expression network of GABA-signaling-related genes**. Genes included in this network are GABA interneuron markers [somatostatin (SST), parvalbumin (PV), neuropeptide Y (NPY), calretinin (CR), calbindin (CALB1), vasoactive intestinal peptide (VIP), cholecystokinin (CCK)], GABA A receptor subunits (alpha 1 (GABRA1), alpha 2 (GABRA2), alpha 5 (GABRA5), and GABA-related genes [GAD67, GAD65, GABA transporter 1 (GAT1)]. Nearness of nodes is proportional to co-expression strength. Along with SST, GAD67, and GAD65, genes in the core module include CALB1, which is often co-localized with SST, and GABRA5, which is postsynaptic to SST-positive GABA neurons.

To assess the biological context of *SST*, we next aimed to identify genes co-expressed with *SST*. In a large cohort of 214 control subjects (Table 2), we identified the top 200 genes positively correlated with *SST* (Table 4). Pathway analysis identified GABA receptor signaling and mitochondrial dysfunction as the top canonical pathways represented (Table 5). Notably, this *SST*-co-regulated gene selection included *GAD67* and *GAD65*. To further assess the specificity of these links, we built a weighted gene co-expression network restricted to GABA-signaling genes (e.g., receptor subunits, GABA neuron markers), which confirmed the tight co-regulation between *SST*, *GAD67*, and *GAD65*, as illustrated by their close proximity in the network graph (Figure 4), demonstrating that they form a tightly linked biological module, and suggesting common upstream regulating factors. Based on these results and on evidence of down-regulation in MDD (20), all further analyses focused on these three genes.

**Table 4:** **Top 200 genes correlated with *SST* in human frontal cortex**.

*ABI2*, *ACOT7*, *ACP1*, *ACTR3B*, *AP2S1*, *APITD1-CORT*, *APOA1BP*, *ARL4C*, *ARPC2*, *ARPC4*, *ATOX1*, *ATP5B*, *ATP5G1*, *ATP5I*, *ATP6V0B*, *ATP6V0D1*, *AZIN1*, *BRP44*, *BSCL2*, *C12orf10*, *C12orf68*, *CA10*, *CACNG3*, *CADM3*, *CALB1*, *CBLN4*, *CDH13*, *CDH8*, *CDK5*, *CDK5R2*, *CHCHD6*, *CHP*, *CISD1*, *CLIP3*, *CMAS*, *COMMD7*, *COPS4*, *CORO1A*, *COX8A*, *CRHBP*, *CRYM*, *CSNK2A1*, *CUTA*, *CYC1*, *DCTN3*, *DCTPP1*, *DDT*, *DLG3*, *DLG4*, *DUSP3*, *EFNB3*, *EIF3K*, *FABP3*, *FKBP1A*, *GABBR2*, *GABRA3*, *GABRA5*, *GABRB3*, *GAD67*, *GAD65*, *GGCT*, *GHITM*, *GLCE*, *GNG3*, *GRIA1*, *GRIK1*, *HMP19*, *HN1*, *HPCA*, *IGF1*, *ITPKA*, *LANCL2*, *LCMT1*, *LPPR4*, *LUZP6*, *MAGED1*, *MAGEH1*, *MAL2*, *MAST3*, *MDH2*, *MFSD4*, *MGST3*, *MIF*, *MMD*, *MRPL28*, *MRPL37*, *MRPL55*, *MRPS24*, *NCAM2*, *NDUFA11*, *NDUFA9*, *NDUFAB1*, *NDUFB5*, *NDUFS3*, *NDUFS6*, *NDUFV1*, *NECAP1*, *NHP2*, *NNAT*, *NPTXR*, *NRIP3*, *NRN1*, *NRSN2*, *NUS1*, *NXPH1*, *OLFM1*, *OXCT1*, *PAFAH1B1*, *PCDH20*, *PGAM1*, *PGAM4*, *PIH1D1*, *PINK1*, *PLD3*, *PNMA1*, *PNOC*, *PPP5C*, *PRAF2*, *PRKCB*, *PSMA5*, *PSMA7*, *PSMB3*, *PSMB5*, *PSMB6*, *PSMB7*, *PSMC3*, *PSMD8*, *PTPRN*, *PTS*, *RAB27B*, *RAB3A*, *RAB3B*, *RAB3C*, *RAB6A*, *RBP4*, *RFK*, *RGS7BP*, *RHBDD2*, *RHEB*, *RNASEK*, *RNF11*, *ROBO2*, *RPH3A*, *SCAMP5*, *SCG2*, *SCG5*, *SCN3B*, *SERINC3*, *SEZ6*, *SEZ6L2*, *SF3B5*, *SLC17A7*, *SLC25A3*, *SLC25A4*, *SLC25A46*, *SLC2A13*, *SLC32A1*, *SLC35B1*, *SNRPD2*, *SPAG7*, *SPHKAP*, *SPINT2*, *SSBP2*, *STK25*, *STMN2*, *SUB1*, *SVOP*, *SYNPR*, *SYT5*, *TAC1*, *TAGLN3*, *TBC1D9*, *TBCB*, *TM9SF2*, *TMEFF2*, *TMEM141*, *TMEM160*, *TMEM203*, *TMEM205*, *TMEM208*, *TMEM59L*, *TMSB10*, *TOLLIP*, *TRAPPC2L*, *TTC9B*, *TUBA1A*, *TUBA4A*, *TUBB*, *TXNL4A*, *UBE2S*, *UQCR11*, *UQCRFS1*, *UQCRH*, *VAMP2*, *VBP1*, *XKR4*, *YWHAG*, *ZCCHC12*, *ZCCHC17*, *ZNF385D*

**Table 5:** **(Top) Mitochondrial dysfunction-related genes with expression correlated with *SST* (14/174 genes) in human frontal cortex. (Bottom) GABA receptor signaling-related genes with expression correlated with *SST* (8/56 genes)**.

Gene symbol	Gene title	Gene accession	Correlation with *SST* (R)
*ATP5B*	ATP synthase, H+ transporting, mitochondrial F1 complex, beta polypeptide	NM_001686	0.759
*COX8A*	Cytochrome *c* oxidase subunit VIIIA (ubiquitous)	NM_004074	0.771
*CYC1*	Cytochrome *c*-1	NM_001916	0.748
*NDUFA9*	NADH dehydrogenase (ubiquinone) 1 alpha subcomplex, 9, 39 kDa	NM_005002	0.742
*NDUFA11*	NADH dehydrogenase (ubiquinone) 1 alpha subcomplex, 11, 14.7 kDa	NM_175614	0.755
*NDUFAB1*	NADH dehydrogenase (ubiquinone) 1, alpha/beta subcomplex, 1, 8 kDa	NM_005003	0.788
*NDUFB5*	NADH dehydrogenase (ubiquinone) 1 beta subcomplex, 5, 16 kDa	NM_002492	0.736
*NDUFS3*	NADH dehydrogenase (ubiquinone) Fe-S protein 3, 30 kDa (NADH-coenzyme Q reductase)	NM_004551	0.735
*NDUFS6*	NADH dehydrogenase (ubiquinone) Fe-S protein 6, 13 kDa (NADH-coenzyme Q reductase)	NM_004553	0.767
*NDUFV1*	NADH dehydrogenase (ubiquinone) flavoprotein 1, 51 kDa	NM_007103	0.754
*PINK1*	PTEN induced putative kinase 1	NM_032409	0.74
*UQCR11*	ubiquinol-cytochrome c reductase, complex	NM_006830	0.735
*UQCRFS1*	ubiquinol-cytochrome c reductase, Rieske	NM_006003	0.812
*UQCRH*	ubiquinol-cytochrome c reductase hinge	NM_006004	0.777

*AP2S1*	adaptor-related protein complex 2, sigma 1 subunit	NM_004069	0.78
*GABBR2*	Gamma-aminobutyric acid (GABA) B receptor, 2	NM_005458	0.75
*GABRA3*	Gamma-aminobutyric acid (GABA) A receptor, alpha 3	NM_000808	0.73
*GABRA5*	Gamma-aminobutyric acid (GABA) A receptor, alpha 5	NM_000810	0.746
*GABRB3*	Gamma-aminobutyric acid (GABA) A receptor, beta 3	NM_021912	0.748
*GAD67*	Glutamate decarboxylase 67 (brain, 67 kDa)	NM_000817	0.736
*GAD65*	Glutamate decarboxylase 65 (pancreatic islets and brain, 65 kDa)	NM_000818	0.791
*SLC32A1*	Solute carrier family 32 (GABA vesicular transporter), member 1	NM_080552	0.773

### Association of *SST*, *GAD67*, and *GAD65* expression with X-chromosome genetic variants in humans

To test the hypothesis of X-chromosome genetic contribution to *SST*, *GAD67*, and *GAD65* gene expression, we performed an eQTL study in the same postmortem dataset. Since *SST*, *GAD67*, and *GAD65* are not located on the X-chromosome, the assumption for an X-chromosome contribution to the expression of those genes is through trans-regulation by X-chromosome-encoded factors (trans-eQTL). After Benjamini Hochberg correction for X-chromosome SNPs (38), 165 X-chromosome SNPs remained significantly associated with expression of *SST*, *GAD67*, and/or *GAD65* (Table 6), identifying 44 X-chromosome transcripts (Table 7). Fifty-nine of these SNPs appeared functional as demonstrated by significant associations with altered expression of these X-transcripts (cis-eQTL; indicated in bold in Table 7). In contrast, two genes chosen for no co-expression with *SST* (*KDM5C* and *C2orf49*) displayed no significant X-chromosome SNPs. Of the 27 Y-chromosome SNPs genotyped, none were associated with gene expression of *SST*, *GAD67*, or *GAD65*. Together, these results “link” *SST*, *GAD67*, and *GAD65* to X-chromosome factors, supporting our central hypothesis of sex chromosome mediated control of GABA-related genes and associated affect regulation. The number of identified SNPs and sexually dimorphic trans-eQTL results (Table 6) suggest that gonadal hormones may play an intermediary role; in other words, our results suggest that gonadal hormones (which are different between males and females) could override or modulate gene expression that is regulated by X-chromosome SNPs.

**Table 6:** **Summary of human frontal cortex (BA11/BA47) eQTL results for females and males**.

	No. of significantSNPs	No. of LD blocks(individual SNPs)	No. of transcriptsidentified
**FEMALES**			
*SST*	87	14 (17)	33
*GAD67*	30	6 (9)	14
*GAD65*	91	15 (21)	16
**MALES**			
*SST*	0	0 (0)	0
*GAD67*	3	0 (3)	7
*GAD65*	19	2 (6)	12

GAD65, glutamate decarboxylase 65; GAD67, glutamate decarboxylase 67; LD, linkage disequilibrium; SNP, single nucleotide polymorphism; SST, somatostatin.

**Table 7:** **X-chromosome genes identified by trans-eQTL association with *SST*, GAD67, or GAD65 in human frontal cortex**.

***FRMPD4***, *CTPS2*, *CALB3*, ***RBBP7***, ***REPS2***, ***PRDX4***, *ACOT9*, *ARX*, ***IL1RAPL1***, ***USP9X***, ***ZNF674***, ***CHST7***, ***SLC9A7***, ***IGBP1***, ***DGAT2L6***, ***PCDH11X***, ***LHFPL1***, ***AMOT***, *SLC6A14*, *CXorf61*, ***IGSF1***, ***MID2***, ***TEX13B***, ***ODZ1***, *SH2D1A*, ***CDKL5***, ***CXorf59***, *PPP1R2P9*, *EDA*, ***ACSL4***, *SMARCA1*, ***OCRL***, *MAGEC2*, *SLITRK4*, *SPANXN1*, *SPRY3*, *WDR40C*, ***WWC3***, ***CLCN4***, *PHEX*, *VSIG1*, *PSMD10*, *ATG4A*, ***COL4A6***

Genes that displayed additional significant cis-eQTL are indicated in bold.

### XY genetic sex decreases GABA-related gene expression

Refer to Table A1 in Appendix for summary of statistical results.

**Figure 5:**
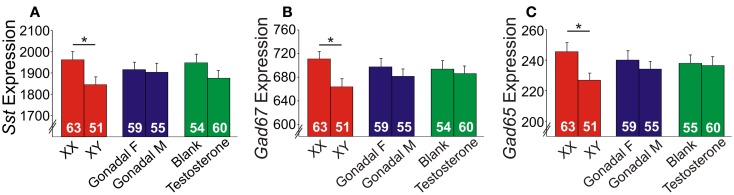
**Expression of GABA-related genes in the frontal cortex of FCG mice**. XY mice had lower **(A)** *Sst*, **(B)** *Gad67*, and **(C)** *Gad65* expression levels compared to XX mice. Error bars indicated mean ± SEM. *p < 0.05. *Y*-axis, truncated arbitrary expression units.

Since it is impossible to separate the role of genetic sex from gonadal sex in humans, as gonadal sex is determined by genetic sex, the X-chromosome trans-eQTL results could reflect indirect hormonal effects. We tested this hypothesis using the FCG mouse model, in which genetic and gonadal sex are artificially decoupled. In this model, the testes-determining gene, *Sry*, was removed from the Y-chromosome and placed on an autosome, hence yielding XX mice with either ovaries or testes, and XY mice with either ovaries or testes (39). We assessed gene expression in the frontal cortex, as this region includes the homolog of the ACC in humans, a corticolimbic region affected in MDD (20, 21). Main factor analyses revealed a significant effect of genetic sex, with reduced expression of *Sst*, *Gad67*, and *Gad65* in XY compared to XX mice, regardless of gonadal sex or adult testosterone exposure (Figures 5A–C). No gonadal sex or testosterone-treatment effects, or interactions between any main factors were observed on expression levels; thus, we represent the results as main factors (Figures 5A–C). Differences in earlier circulating testosterone levels in XX and XY mice (i.e., before gonadectomy) were unlikely to explain the differences, as prior studies report no peripubertal hormone differences (40) or adult testosterone level differences in FCG mice (41, 42). These results confirm in mice the contribution of genetic sex to *Sst*, *Gad67*, and *Gad65* expression suggested by the human studies. However, the lower expressions in genetic male mice were surprising since our human findings indicated low *SST* in women with MDD, suggesting that the effect of male genetic sex may be opposed by other factors in “intact” human males, potentially testosterone, since prior studies suggest that it can lower anxiety (43).

### Anxiety-like behaviors are increased by XY genetic sex and decreased by circulating testosterone in mice

**Figure 6:**
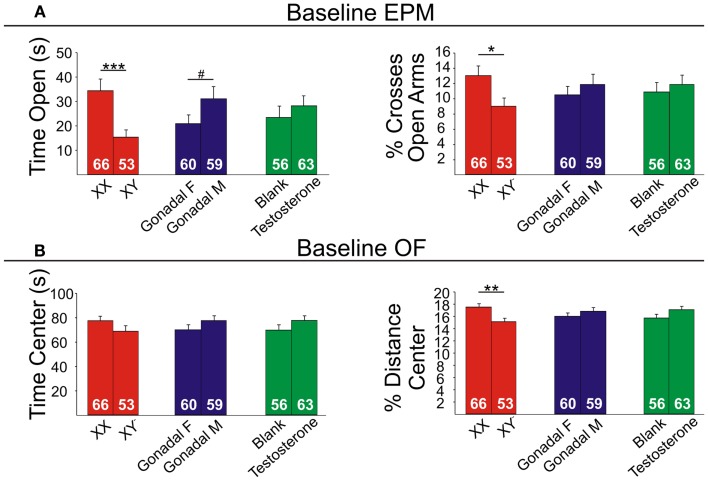
**Baseline emotionality measures in FCG mice. (A)** Baseline elevated plus maze (EPM) results for time (left) and percent crosses (right) into open arms. **(B)** Baseline open Field (OF) results for time (left) and percent distance (right) in the center. Numbers at the base of bars indicate group sizes. Error bars indicated mean ± SEM. ***p < 0.001, **p < 0.01; *p < 0.05; #*p* < 0.1.

**Figure 7:**
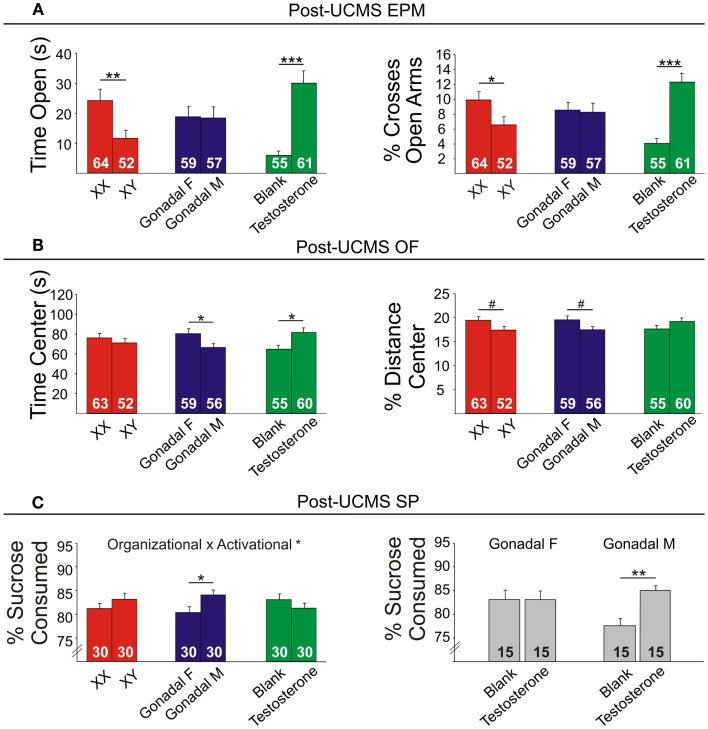
**Post-UCMS emotionality measures in FCG mice**. **(A)** Post-UCMS elevated plus maze (EPM) results for time (left) and percent crosses (right) into open arms. **(B)** Post-UCMS open Field (OF) results for time (left) and percent distance (right) in the center. **(C)** Post-UCMS sucrose preference (SP) results for percent sucrose consumed for all groups (left) and for the organizational × activational interaction (right). Numbers at the base of bars indicate group sizes. Error bars indicate mean ± SEM. ***p < 0.001, **p < 0.01; *p < 0.05; #*p* < 0.1.

**Figure 8:**
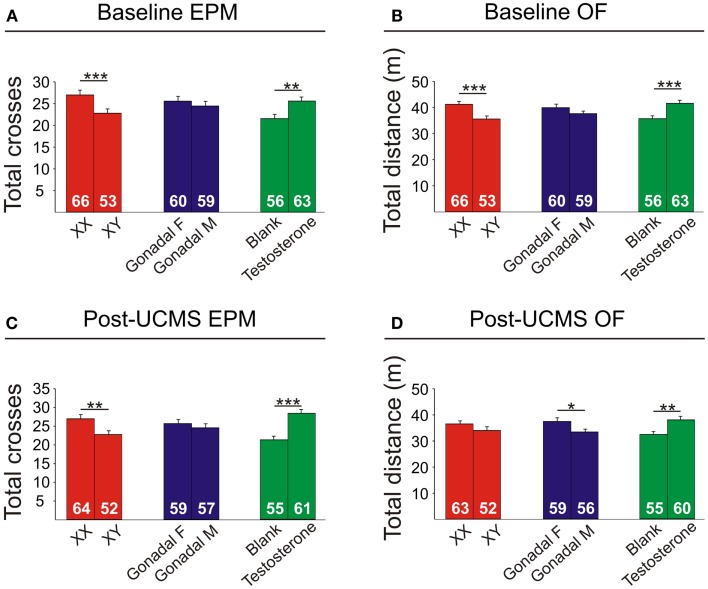
**Locomotor activity measures in FCG mice**. Elevated plus maze (EPM) results for total crosses at baseline **(A)** and post-UCMS **(C)**. Open field (OF) results for total distance at baseline **(B)** and post-UCMS **(D)**. Numbers at the base of bars indicate N. Error bars indicate mean ± SEM. ***p < 0.001; **p < 0.01; *p < 0.05.

Refer to Table A2 in Appendix for summary of statistical results. To investigate whether testosterone could play a compensatory role to lower anxiety in males, we analyzed anxiety-/depressive-like behavior in FCG mice under baseline “non-stress” conditions and after exposure to UCMS. UCMS is a behavioral paradigm that robustly increases behavioral emotionality, thus presenting some homologous features associated with human depression (although not “modeling” this complex human disorder, but providing critical information about response to chronic stress) (27). There were no significant interactions of main factors in the EPM or OF, and results are displayed as main effects (Figures 6 and 7). These measures were controlled for locomotor activity, which independently displayed effects of sex-related factors (see below; Figure 8).

#### Genetic sex

Contrary to our prediction, but consistent with reduced expression of *Sst*, *Gad67*, and *Gad65* (Figure 5), XY mice, regardless of gonadal sex or adult testosterone exposure, consistently exhibited higher anxiety-like behaviors than XX mice. In the EPM at baseline, XY mice spent significantly less time (Figure 6A left) and had lower percent crosses into the aversive open arms (Figure 6B right) than XX mice. Baseline results in the OF were consistent with the EPM results, with XY mice exhibiting lower percent distance in the aversive center of the OF (Figure 6B right) than XX mice, together providing converging evidence indicative of elevated anxiety-like behaviors in XY mice at baseline. Behavioral results after prolonged exposure to UCMS were consistent with baseline results. XY mice spent less time in the open arms (Figure 7A left), had lower percent crosses into the open arms of the EPM (Figure 7A right), and had trend-level lower percentage distance in the center of the OF (Figure 7B right). There was no main effect of genetic sex on percent sucrose consumed in the sucrose preference test (Figure 7C left).

#### Adult testosterone-treatment

As predicted, mice exposed to the activational effects of testosterone in adulthood (similar to levels of a normal male) exhibited lower adult anxiety-like behavior, regardless of genetic sex or gonadal sex. At baseline, mice treated with testosterone had trend-level elevated percent distance in the center of the OF (Figure 6B right). The activational effects of testosterone were more robust after UCMS. Testosterone-treated mice spent more time (Figure 7A left) and had higher percent crosses into the open arms of the EPM (Figure 7A right), and spent more time in the center of the OF (Figure 7B left). There was no main effect of circulating testosterone on percent sucrose consumed in the sucrose preference test (Figure 7C left).

#### Gonadal sex

Gonadal sex had inconsistent effects on anxiety-like behavior: mice with testes (and therefore testosterone) during development (gonadal males) exhibited a trend for more time in the open arms of the EPM at baseline (Figure 6A left; lower anxiety-like behavior). After UCMS, gonadal males showed a trend for less time and percent distance in the center of the OF (Figure 7B; higher anxiety-like behavior) than gonadal females. While gonadal sex had minimal effects on anxiety-like behavior, it was the only main factor influencing anhedonia-/depressive-like behavior, as gonadal males exhibited increased sucrose preference (Figure 7C left). In line with the well-established concept that organizational effects during development create differences in how the adult brain responds to hormones (39), testosterone decreased anhedonia-/depressive-like behavior in the sucrose preference test in gonadal males (Figure 7C right); this result is consistent with the frequent observation that gonadal males and females respond differently to adult hormone manipulations [e.g., (9, 44)], indicating that hormone-treatments that influence mood in men could affect women differently.

### Locomotor activity is decreased by XY genetic sex and increased by circulating testosterone

Refer to Table A3 in Appendix for summary of statistical results. There were no significant interactions of main factors in the EPM or OF, and results are displayed as main effects (Figure 8).

#### Genetic sex

XY mice exhibited lower baseline activity than XX mice, as indicated by fewer total crosses the EPM (Figure 8A) and less total distance traveled in the OF (Figure 8B). After chronic stress, XY mice had fewer total crosses in the EPM (Figure 8C), consistent with reduced activity.

#### Adult testosterone-treatment

Testosterone-treatment in adulthood resulted in increased baseline activity, with testosterone-treated mice having more crosses in the EPM (Figure 8A) and more total distance traveled in the OF (Figure 8B). Testosterone-treated mice also had higher activity after UCMS, with greater total EPM crosses (Figure 8C) and more total distance traveled in the OF (Figure 8D).

#### Gonadal sex

Gonadal sex had a slight effect on activity, as gonadal males exhibited lower OF total distance after UCMS than gonadal females (Figure 8D).

## Discussion

Using gene array meta-analysis, we identified a sex-specific effect on decreased expression of *SST* in the postmortem brain of MDD patients, with depressed women exhibiting more robust reduction in *SST* gene expression (Figures 2 and 3). We then used a gene network approach to investigate genes co-expressed with *SST*, and identified GABA receptor signaling as a top pathway represented by *SST*-co-expressed genes. Based on the tight co-expression between *SST*, *GAD67*, and *GAD65* (Figure 4), and on evidence for changes in expression in MDD (20), we focused our investigations on those three genes in subsequent analyses. We performed an eQTL study in human postmortem brains to investigate potential mechanisms underlying the female-specific decrease in GABA-related gene expression in MDD, and found multiple SNPs on the X-chromosome significantly associated with *SST*, *GAD67*, and/or *GAD65* expression differences. Using the FCG mice to dissect the respective contributions of sex-related factors, we then show that genetic sex (i.e., X/Y-chromosome factors), independent of gonadal sex or circulating testosterorone, influences expression of these same genes in pro-anxiety/depression directions (lower expression in XY) (Figure 5), suggesting a mouse/human conserved mechanism of *SST*, *GAD67*, and *GAD65* trans-regulation by X/Y-encoded genes. Notably, XY mice also demonstrated elevated anxiety-like behaviors (Figures 6 and 7). Treating mice with adult circulating testosterone levels similar to a normal male had the expected opposite effect of lowering anxiety-like behaviors, but had surprisingly no-effect on GABA-related gene expression. Thus, male genetic sex increased anxiety-like behaviors, while treatment with male-like levels of testosterone decreased anxiety-like behaviors.

### Sexual dimorphism in low *SST* in MDD

Previous studies showed decreased *SST* expression in MDD (16, 20–22), with potential greater effects in women with depression (21). Using a meta-analysis and meta-regression for sex effect, we confirmed that *SST* reduction is significantly more robust in female MDD (Figures 2 and 3), mirroring the increased female vulnerability to MDD. *SST*-positive GABA interneurons preferentially target the dendritic compartment of pyramidal neurons and contribute to sensory integration and fine-tuning of incoming information onto pyramidal cells. Combined with results indicating that genetic sex affects *SST*, *GAD67*, and *GAD65* gene expression, these findings suggest that genetic sex is a moderating biological factor influencing the function of a GABA-related module. Reduced *SST* may be a marker of reduced GABA-mediated dendritic inhibition, the end result being increased activation of targeted pyramidal cells. As a hub in the corticolimbic network of affect regulation, the sgACC consistently shows elevated metabolic activity with the induction of depressive state (45, 46), with metabolic activity returning to normal following antidepressant treatment (46) or deep brain stimulation (47). It should be noted that elevated sgACC activation is not limited to mood disorders, nor is the sgACC the only region exhibiting altered activation in mood disorders; however, this elevated activation and normalization after different modalities of treatment has been consistently shown across many studies [reviewed in (48)]. Interestingly, evidence suggests that some features of sgACC dysfunction in MDD are sexually dimorphic (49). Hence, reduced GABA-mediated inhibition onto pyramidal dendrites may underlie the increased sgACC activation reported in MDD patients (45) and restoring dendritic inhibitory function may reduce pyramidal cell activation and contribute to reduced sgACC activation with positive treatment response (46, 47). Whether such approaches may be more effective in female subjects with MDD is not known. We recently showed that expression of *Sst*, *Gad67*, and *Gad65* is dependent upon brain-derived neurotrophic factor (BDNF) signaling (16, 20), and reports also indicate sexually dimorphic effects of reduced BDNF signaling on anxiety-/depressive-like behaviors (50–52). Combined with evidence that GABA-signaling influences emotionality (18), it is possible that the sexually dimorphic effect of BDNF signaling on anxiety-depressive-like behavior could be mediated through effects on GABA-related gene expression.

### *SST* gene network

Pathway analysis of the top *SST*-co-expressed genes confirmed its contribution to GABA-signaling and also identified mitochondrial function as the top associated pathway. Of note, we reported sex differences in expression of genes related to mitochondrial function in the amygdala of mice and humans (53). Thus, sex-dependent differences in mitochondrial function may represent a potential mediating factor in the male/female differential effects of MDD in *SST* cells. Interestingly, we recently found that *GAD67* and *GAD65* are reduced in the sgACC of male patients with MDD, but not female patients (20). Combined with our present results indicating a role for XY genetic sex in decreasing *Gad65/67* in a homologous brain region in the mouse, these results imply that males may be more likely to have reduced expression of *GAD65*/*67* in the ACC due to genetic sex effects. Interestingly, *GAD65/67* and GABA-related changes were prominent the amygdala of females with MDD (16) and not changed in the amygdala of males with MDD (54), demonstrating both sex- and brain region-specific contributions to GABA-related MDD pathology.

### Association of X-chromosome polymorphisms and expression of GABA-related genes

Since *SST*, *GAD67*, and *GAD65* are not located on X-chromosomes, the trans-eQTL results showing association of these genes with X-chromosome SNPs suggest that the X-chromosome may contain genes that function as upstream regulators of GABA gene expression. The large numbers of SNPs with significant trans-eQTL suggest that these indirect pathways may be complex and include multiple genes and factors. Interestingly, one of the associated X-chromosome genes was *FRMPD4*, a positive regulator of dendritic spine density and morphology, suggesting that *FRMPD4* may influence expression of GABA-related genes through shared downstream biological function related to neuronal signaling. Another identified X-chromosome gene was *USP9X*, a gene that escapes X-inactivation that has been linked to synaptic development (55). *Usp9x* expression was also reported to be higher in genetic female mice compared to genetic males, regardless of hormone-treatment (56). Of note, the SNPs that identified *FRMPD4* and *USP9X* were associated with expression of these genes (cis-eQTL) only in females, suggesting that genetic variation at these SNPs affect females only, potentially through sex-specific variations in transcription factor complexes. Alternatively, gonadal hormones could play an intermediary role, either masking or enhancing genetic sex mediated effects. In contrast to these X-chromosome indirect effects, circulating testosterone was not associated with *Sst*, *Gad67*, or *Gad65* expression in FCG mice. Hence our results support a contribution of genetic sex to sexual dimorphism in affect dysregulation in human subjects, potentially mediated by X-chromosome trans-regulation of key GABA-related genes.

### Genetic sex as a contributing factor

The origin of the effect of genetic sex on GABA-related gene expression and associated anxiety-like behavior is currently not known. Sex chromosomes differ between males and females in the presence or absence of a Y and the dosage of X-chromosomes. A role for Y-chromosome genes (other than *Sry*) would be consistent with reported effects of the Y-chromosome on male reproduction (57). Due to random X-inactivation, female tissues are mosaics, with about half of cells expressing an active maternal X and the other half expressing an active paternal X. Thus, if a maternally imprinted X causes increases in gene expression, XX individuals would have overall lower expression due to the presence of the paternal imprint in half of the cells. Also, a number of genes escape X-inactivation (∼15% in humans and ∼3% in mice, depending on tissue; (58)), and evidence suggests that “X-escapees” in females could contribute to sexual dimorphism. For instance, X-chromosome number causes sex differences in dorsal striatal gene expression (59) and male sex behavior (60) in mice. Mice that are haploinsufficient for genes escaping X-inactivation (i.e., XO mice) have higher levels of fear reactivity compared to XX mice (61). In women with fragile X or Turner Syndrome (XO), X-haploinsufficiency is associated with elevated arousal under stressful situations (62). These results from mice and humans with X-haploinsufficiency mirror our finding of elevated anxiety-like behavior in XY FCG mice, suggesting that differences in X-dosage between genetic male and female mice may contribute to the genetic sex effect on behavioral emotionality reported here. Follow-up studies in XO and XXY mice may determine if observed genetic sex effects on anxiety-like behavior and gene expression are due to X- or Y-linked genes.

### Opposing effects of XY genetic sex and circulating testosterone on anxiety

Although reported for the first time for anxiety-like behavior, one conventional interpretation in the field of sexual dimorphism is that male genetic sex exerts a compensatory effect to reduce behavioral differences otherwise induced by circulating testosterone (63). Importantly, other studies using FCG mice have reported opposing actions of male genetic sex and male circulating hormones. For instance, circulating testosterone has an inhibitory effect, and XY genetic sex has a stimulatory effect on immune response (42). Similarly, circulating testosterone increased, and XY genetic sex decreased the expression of male sex behavior (60). Thus, the pattern of results we report for anxiety-like behaviors supports a general hypothesis for a compensatory mechanism between male genetic sex and testosterone, serving to decrease differences between males and females (63). Since normal “intact” male mice exhibit lower emotionality than females (64) and since we observed a more robust effect of treatment with male-like levels of testosterone on lowering anxiety-like behaviors compared to the anxiogenic contribution of genetic sex (Figure 7), circulating testosterone seems to “win out” in a normal, intact male; the end result being males displaying lower anxiety-like behavior. XY genetic male sex also had decreased locomotor activity (Figure 8), while circulating testosterone had a similar pattern of opposing this effect. Note that despite controlling the measures of anxiety-like behaviors for locomotor activity, opposite patterns are frequently observed and often difficult to dissociate in rodents (32). QTL mapping studies have identified chromosomal regions associated with both anxiety-like behavior and locomotor activity (65), suggesting epistatic regulation of emotionality and activity downstream from common genes.

### Limitations

Our gene co-expression and eQTL studies were performed in a large cohort of control subjects due to the limited number of postmortem MDD subjects available for genetic analysis. Since we had an *a priori* hypothesis for sexual dimorphism, we performed eQTL analysis only for the X and Y-chromosomes; future unbiased genome-wide studies may yield additional findings.

FCG mice provide a practical tool for parsing relative contributions of genetic sex, gonadal sex, and circulating hormones to sexual dimorphism, although there are limitations to this model: first, XY^−^ mice are not identical to wild-type XY mice, but XY^−^*Sry* male mice are fertile and display no differences from wild-types in testosterone levels, and serve as the closest equivalent to “normal” males (40–42). Second, since *Sry* is also expressed in the adult mouse brain (66), it may be impossible to separate *indirect* (through testes development) and *direct* (through brain gene expression) effects of *Sry*. Thus, differences between mice with and without *Sry* could be due to direct *Sry* effects. Additionally, gene expression could be disrupted after autosomal *Sry* transgene insertion; although no correlation was observed between *Sry* and gene expression. Third, since mice are GDX in adulthood, this experimental design does not include normal, intact mice. Finally, genetic and behavioral results in mice may not translate to humans; however we show that female mice are more susceptible to chronic stress-induced high emotionality (64), suggesting the approach is appropriate to investigate biological mechanisms underlying sexual dimorphism in vulnerability to mood disorders.

## Summary

In conclusion, these studies provide evidence for more robust down-regulation of *SST* in women compared to men with MDD, and suggest a link between X-chromosomes and sexual dimorphism in mood disorders. This link is supported by studies in FCG mice demonstrating a role for genetic sex in control of *Sst*, *Gad67*, and *Gad65* expression, with concomitant effects on behavioral emotionality. Together, these results support genetic sex as a contributing factor influencing an MDD-related biological module related to GABA function and mood-related behaviors. Notably, since related *SST*/GABA changes are observed in other psychiatric and neurodegenerative disorders, this novel finding may have broader implications for general risk for adult psychopathology that is linked to genetic sex and GABA function.

## Conflict of Interest Statement

David A. Lewis currently receives investigator-initiated research support from Bristol-Myers Squibb and Pfizer, and in 2010–2012 served as a consultant in the areas of target identification/validation and new compound development to Bristol-Myers Squibb and Concert Pharmaceuticals. The other co-authors declare that the research was conducted in the absence of any commercial or financial relationships that could be construed as a potential conflict of interest.

## Appendix

### Methods

#### Meta-analysis of *SST* gene expression in MDD datasets

##### Gene array data pre-processing

Microarrays were scanned and summarized by manufacturers’ defaults. Data from Affymetrix arrays were processed by robust multi-array (RMA) method and data from Illumina arrays by manufacturer’s BeadArray software for probe analysis. Batch effects were evaluated and normalized. Oligonucleotide probes (or probesets) were matched to gene symbols using hgu133plus2.db and illuminaHumanv4.db Bioconductor packages.

##### Meta-analysis using random effect model

Datasets and meta-analysis methods and results were described previously (36, 37). Briefly, we adopted linear models to account for potential confounding covariates and applied a meta-analysis pipeline to combine eight studies for identification of MDD-associated genes. Note that some studies were performed in different brain regions of the same subjects. To account for this putative dependence structure among studies from the same patients, we kept the same permutation order for each pair of studies of the same cohort in the permutation analysis of individual studies. We then used a random effects model (REM) to detect changes in *SST* expression by combining gene effects across all studies.

##### Meta-regression with variable selection (MetaRG_BIC)

We develop a novel meta-regression framework to investigate across studies differential effect sizes potentially associated with study-level variables (i.e., sex/gender; Table 1). Unlike meta-analysis in clinical or epidemiological research where large numbers of studies may be available to create a meta-regression model, only a small number (e.g., 5–15) of studies are available in a common microarray meta-analysis. Since only a very small number (e.g., 0–1) of variables contribute to the differential effect sizes for a given gene, we used a variable selection approach here to select appropriate covariates to use for *SST*. Specifically, all possible meta-regression models that include at most one (0 or 1) study-specific variables are computed and compared. The following meta-regression model with variable selection is considered:

$$\text{MetaRG }\left(w_{\text{g}}\right):\;\;\;Y_{\text{gk}}=\mu_{\text{g}}+\sum_{\text{l}=1}^{\text{L}}w_{g1}*\beta_{{\text{g}}_{1}}*X_{\text{lk}}+\alpha_{\text{gk}}+\eta_{\text{gk}},$$

where *Y*_gk_ is the effect size for gene g and study k, *X*_lk_ is the value for study-level variable *l* in study *k*, w_gl_ is the 0–1 weight assigned to the lth study-level variable (*L* = 3) and we restrict *w*_gl_ such that at most one study-level variable is selected $\left(\sum_{l}w_{\text{gl}}\leq 1\right)$. We denote by BIC(*w*_g_) as the Bayesian Information Criterion value associated with the meta-regression model MetaRG(*w*_g_) and we search for the best-fit model with the smallest BIC value by ${\text{w}}_{g}^{*}=\left(w_{\text{g1}}^{\text{*}},\;w_{\text{g2}}^{\text{*}},\;...,\;w_{\text{gL}}^{\text{*}}\right)=\arg\;\min_{\text{w}\in \text{W}}\text{ BIC(w)}$. Based on the selected model ${\text{MetaRG}(\text{w}}_{g}^{*}\text{),}$ likelihood ratio test is applied to test for the β_gl_ with non-zero ${\text{w}}_{\text{gl}}^{*}$ (*H*_0_: β_gl_ = 0 versus *H*_A_: β_gl_ ≠ 0) and derive the *p*-value of gene g. This added variable selection avoids including more than one study-specific variable in the model and allows assessment of biomarkers related to different study-specific variable (e.g., gene A might have differential effect size in sex while gene B might have differential effect size in brain region or platform), which biologically gives a more appealing conclusion and interpretation. These derived *p*-values are, however, not the real *p*-values for DE gene detection since they are biased from the variable selection procedure. As a result, we perform a conventional genome-wide permutation test that randomly permutes the disease labels within each MDD-control pair to generate a null distribution of individual study effect sizes. We then repeat the above variable selection procedure to derive the null distribution under permutation analysis and assess the meta-regression *p*-value of each gene.

##### Forest plots for visualization of meta-analysis findings

A forest plot is a graphical display designed to illustrate the relative strength of disease effects across multiple studies addressing the same question. The plot shows if the overall effect is based on many studies or a few, on studies that are precise (study with small variance) or imprecise (study with large variance), and whether the effects for all studies tend to line up, or if they vary substantially from one study to the next. The plot can also highlight anomalies and outliers.

#### Gene networks and expression quantitative trait loci

##### Gene arrays and expression

Total RNA was extracted from frozen BA11 and BA47 samples stored in TRIZOL and were processed for microarray analysis using GeneChip Human Gene 1.1 ST from Affymetrix according to manufacturer’s protocol (http://www.affymetrix.com). Gene expression data was extracted using Expression Console build 1.2.1.20. The normalization method is based on quantile normalization to eliminate batch effects. Data from arrays were processed by RMA method. Gene expression probes were processed at gene-level and taken in log2 scale for further analysis. After normalization, 33,297 gene-level probes remained.

##### Genotyping

All 214 control samples were run on Affymetrix SNP 6.0 arrays for genotyping. Among the 214 samples, 10 RNA samples did not pass quality control. Genotype calls were generated using Affymetrix Genotyping Console version 4.1.3. For intensity quality control, we used Contrast QC, which is the per sample QC test in the Affymetrix SNP 6.0 intensity array; two samples were removed after QC. Birdseed v2 algorithm was used for genotyping, using EM algorithm to drive maximum likelihood fit of two dimensional Gaussian mixture model.

##### Trans-eQTL mapping

Single nucleotide polymorphisms were filtered out using the following criteria: (i) sample missing rate greater than 10%, (ii) Minor allele frequency (MAF) less than 5%, and (iii) *p*-value of Hardy-Weinberg equilibrium (HWE) test less than 10^−3^ (females only). 24,296 X-chromosome SNPs were included. In the eQTL model, we adjusted for age, pH, and RIN values (these three covariates were significantly associated with gene expression in both BA11 and BA47) since the effects of those covariates may confound eQTL findings. The eQTL model with three covariates for a given genotype:

$$G_{\text{ij}}=\alpha +\gamma X_{j}+\sum_{\text{k}=1}^{\text{3}}\;\beta_{\text{k}}{\text{S}}_{\text{jk}}+\in_{\text{ij}}$$

G_ij_: gene expression of gene *i* of subject *j*
γ_i_: effect of the selected genotype to gene i based on additive model
*X_j_*: genotype of subject *i*,0 (homozygous major alleles), 1 (heterozygous calls), and 2 (homozygous minor alleles).
β*_ik_*: the effect of covariates *k; k* = 1 (age), 2 (pH), and 3 (RIN) in gene *i*
*S_jk_*: the value of covariate *k* of subject *j*; *k* = 1 (age), 2 (pH), and 3 (RIN)

$$\in_{\text{ij}}∼N\left(0,1\right)$$

We used the “Matrix eQTL” R package, a recent computationally efficient package for eQTL analysis (67), to detect the desired trans-eQTLs.

We applied a meta-analysis method called adaptive weighted (AW) Fisher’s method to combine the *p*-values of eQTLs from two brain regions (BA11 and BA47), since the correlation between the expression of BA11 and BA47 are high (68). The AW Fisher’s method assigns different weights (0 or 1) to each eQTL finding from two brain regions; the testing statistics $\chi_{\text{AW}}^{2}=-\sum_{k=1}^{2}W_{\text{k}}\cdot \log\left(P_{\text{k}}\right)$, *w*_k_ = 0 or 1, and it searches through all possible weights to achieve the best adaptive weight with the smallest *p-*value. In order to avoid heterogeneity of eQTL finding across brain regions, we selected only eQTLs that were identified in both brain regions in the AW Fisher’s method. The Benjamini Hochberg procedure was used to control false discovery rate (FDR) of multiple eQTLs (38). By setting *q* value 0.05, we detected 87, 30, and 91 trans-eQTLs of genes *SST*, *GAD67*, and *GAD65* in female subjects, respectively; For male subjects, 0, 3, and 20 trans-eQTLs were detected from genes *SST*, *GAD67*, and *GAD65*, respectively.

##### Linkage disequilibrium mapping of significant X-chromosome SNPs

Linkage disequilibrium (LD) is the correlation between genotypes at different loci in a population. We can use *D* to quantify LD, which measures how far off genotype frequencies are from the expectation under no LD. For example, suppose two alleles are A_1_, A_2_, B_1_, B_2_, and the frequencies are *p_1_*, *p_2_*, *q_1_*, *q_2_*. *P_11_* is the frequency of A_1_B_1_ gametes, *P_12_* is the frequency of A_1_B_2_ gametes, and so on. Define *D* = *P_11_* – *p_1_ q_1_*, D = 0 if there is no LD. We used *D*′ = *D*/*D*_max_, where

$$D_{\max}=\left\{\begin{array}{cc} \max\;\left(-p_{1}q_{1}-p_{2}q_{2}\right),\; & \text{when D < 0},\\ \min\;\left(p_{1}q_{2},\;p_{1}q_{2}\right),\;\; & \text{when D > 0} \end{array}\right.$$

Alternatively, we can use correlation coefficient *r*^2^, which can be calculated by

$$r^{2}=\frac{D^{2}}{p_{1}p_{2}q_{1}q_{2}}$$

The “Haploview” software was used for visualization of D′ (69).

#### Real-time quantitative PCR

Total RNA was extracted from tissue punches using the Allprep^®^ DNA/RNA Micro Kit (Qiagen, Valencia, CA, USA) and assessed using chromatography (Agilent Bioanalyzer; Santa Clara, CA, USA). RIN was 8.35 ± 0.04 (mean ± SEM), indicating excellent RNA quality. One-hundred nanograms of total RNA was reverse transcribed into cDNA using QScript cDNA Supermix [oligo(dT) and random primers (Quanta Biosciences, Gaithersburg, MD, USA)]. Small PCR products (70–100 base pairs) were amplified on a Mastercycler^®^ ep *Realplex*^2^ qPCR machine (Eppendorf, Hamburg, Germany), using universal PCR conditions [65–59°C touch-down and 40 cycles (10 s at 95°C, 10 s at 59°C, and 10 s at 72°C)]. cDNA was amplified in 15 μl reactions [0.1X SYBR-green, 3 mM MgCl2, 200 nM dNTPs, 200 nM primers, 0.25 unit Platinum Taq DNA polymerase (Invitrogen, Carlsbad, CA, USA)]. Primer-dimers were assessed by amplifying primers without cDNA. Primers were retained if they produced no primer-dimers or non-specific signal only after 35 cycles. Samples were run in quadruplicate and results were calculated as the geometric mean of relative intensities compared to two internal controls (actin and glyceraldehyde-3-phosphate dehydrogenase). Results are expressed as the amount of arbitrary signal (2^−dCT^ × 10,000).

**Table A1:** **Statistical values associated *Sst*, *Gad67*, and *Gad65* gene expression in the frontal cortex of FCG mice**.

Dependent measure	Main genetic effect	Main activational effect	Main organizational effect
*Sst*	***F* **=** 4.25; df **=** 1; *p* **<** 0.045**	*F* = 0.08; df = 1; *p* > 0.75	*F* = 1.90; df = 1; *p* > 0.15
*Gad67*	***F* **=** 5.91; df **=** 1; *p* **<** 0.02**	*F* = 0.53; df = 1; *p* > 0.45	*F* = 0.30; df = 1; *p* > 0.55
*Gad65*	***F* **=** 5.61; df **=** 1; *p* **<** 0.025**	*F* = 0.51; df = 1; *p* > 0.45	*F* = 0.04; df = 1; *p* > 0.80

Gad65, glutamate decarboxylase 65; Gad67, glutamate decarboxylase 67; Sst, somatostatin. Text in bold indicates statistically significant results.

**Table A2:** **Statistical values associated with baseline (top) and post-UCMS (bottom) emotionality measures in FCG mice**.

	Dependent measure	Main genetic effect	Main organizational effect	Main activational effect
Baseline	Time in open arms (EPM)	***F***** **=** 10.11**; **df **=** 1**; ***p***** **<** 0.002**	*F* = 2.80; df = 1; *p* < 0.1	*F* = 0.97; df = 1; *p* > 0.3
	% Crosses into open arms (EPM)	***F***** **=** 5.58**; **df **=** 1**; ***p***** **<** 0.03**	*F* = 0.60; df = 1; *p* > 0.4	*F* = 0.16; df = 1; *p* > 0.6
	Time in center (OF)	*F* = 1.89; df = 1; *p* > 0.1	*F* = 2.16; df = 1; *p* > 0.1	*F* = 1.95; df = 1; *p* > 0.1
	% Distance in center (OF)	***F***** **=** 8.16**; **df **=** 1**; ***p***** **<** 0.01**	*F* = 1.58; df = 1; *p* > 0.2	*F* = 2.70; df = 1; *p* > 0.1
Post-UCMS	Time in open arms (EPM)	***F***** **=** 7.93**; **df **=** 1**; ***p***** **<** 0.007**	*F* = 0.20; df = 1; *p* > 0.6	***F***** **=** 27.30**; **df **=** 1**; ***p***** **<** 0.00001**
	% Crosses into open arms (EPM)	***F***** **=** 5.48**; **df **=** 1**; ***p***** **<** 0.03**	*F* = 0.09; df = 1; *p* > 0.7	***F***** **=** 32.67**; **df **=** 1**; ***p***** **<** 0.00001**
	Time in center (OF)	*F* = 0.45; df = 1; *p* > 0.5	***F***** **=** 4.19**; **df **=** 1**; ***p***** **<** 0.05**	***F***** **=** 6.35**; **df **=** 1**; ***p***** **<** 0.02**
	% Distance in center (OF)	*F* = 3.35; df = 1; *p* < 0.08	*F* = 3.31; df = 1; *p* < 0.08	*F* = 1.70; df = 1; *p* > 0.1
	% Sucrose consumed (SP)^1^	*F* = 1.22; df = 1; *p* > 0.25	***F***** **=** 6.42**; **df **=** 1**; ***p***** **<** 0.02**	*F* = 1.56; df = 1; *p* > 0.20

^1^There was a significant interaction between organizational and activational effects on percent sucrose consumed (*F* = 5.62; df = 1; *p* < 0.025).EPM, elevated plus maze; OF, open field; SP, sucrose preference. Text in bold indicates statistically significant results.

**Table A3:** **Statistical values associated with baseline (top) and post-UCMS (bottom) activity measures in FCG mice**.

	Dependent measure	Main genetic effect	Main organizational effect	Main activational effect
Baseline	Total crosses (EPM)	***F***** **=** 14.12**; **df **=** 1**; ***p***** **<** 0.001**	*F* = 1.12; df = 1; *p* > 0.2	***F***** **=** 9.21**; **df **=** 1**; ***p***** **<** 0.005**
	Total distance (OF)	***F***** **=** 12.86**; **df **=** 1**; ***p***** **<** 0.001**	*F* = 1.21; df = 1; *p* > 0.2	***F***** **=** 13.28**; **df **=** 1**; ***p***** **<** 0.001**
Post-UCMS	Total crosses (EPM)	***F***** **=** 7.85**; **df **=** 1**; ***p***** **<** 0.007**	*F* = 0.094; df = 1; *p* > 0.7	***F***** **=** 24.66**; **df **=** 1**; ***p***** **<** 0.00001**
	Total distance (OF)	*F* = 1.70; df = 1; *p* > 0.1	***F***** **=** 4.12**; **df **=** 1**; ***p***** **<** 0.05**	***F***** **=** 8.97**; **df **=** 1**; ***p***** **<** 0.005**

EPM, elevated plus maze; OF, open field. Text in bold indicates statistically significant results.
